# Immunological targeting of tumor cells undergoing an epithelial-mesenchymal transition via a recombinant brachyury-yeast vaccine

**DOI:** 10.18632/oncotarget.1295

**Published:** 2013-09-26

**Authors:** Duane H. Hamilton, Mary T. Litzinger, Alessandra Jales, Bruce Huang, Romaine I. Fernando, James W. Hodge, Andressa Ardiani, David Apelian, Jeffrey Schlom, Claudia Palena

**Affiliations:** ^1^ Laboratory of Tumor Immunology and Biology, Center for Cancer Research, National Cancer Institute, National Institutes of Health, Bethesda, Maryland; ^2^ GlobeImmune Inc., Louisville, Colorado

**Keywords:** epithelial-mesenchymal transition, brachyury, T-box transcription factor, cancer vaccine, T-cell immunotherapy

## Abstract

The embryonic T-box transcription factor brachyury is aberrantly expressed in a range of human tumors. Previous studies have demonstrated that brachyury is a driver of the epithelial-mesenchymal transition (EMT), a process associated with cancer progression. Brachyury expression in human tumor cells enhances tumor invasiveness *in vitro* and metastasis *in vivo*, and induces resistance to various conventional therapeutics including chemotherapy and radiation. These characteristics, and the selective expression of brachyury for a range of human tumor types vs. normal adult tissues, make brachyury an attractive tumor target. Due to its intracellular localization and the “undruggable” character of transcription factors, available options to target brachyury are currently limited. Here we report on the development and characterization of an immunological platform for the efficient targeting of brachyury-positive tumors consisting of a heat-killed, recombinant *Saccharomyces cerevisiae* (yeast)–brachyury vector-based vaccine (designated as GI-6301) that expresses the full-length human brachyury protein. We demonstrate that human dendritic cells treated with recombinant yeast-brachyury can activate and expand brachyury-specific CD4^+^ and CD8^+^ T cells *in vitro* that, in turn, can effectively lyse human tumor cells expressing the brachyury protein. Vaccination of mice with recombinant yeast-brachyury is also shown here to elicit brachyury-specific CD4^+^ and CD8^+^ T-cell responses, and to induce anti-tumor immunity in the absence of toxicity. Based on these results, a Phase I clinical trial of GI-6301 is currently ongoing in patients with advanced tumors; to our knowledge, this is the first vaccine platform aimed at targeting a driver of tumor EMT that has successfully reached the clinical stage.

## INTRODUCTION

The T-box transcription factor T, also known as brachyury, was initially identified as a protein required for murine embryonic mesodermal development [1, 2], which involves the conversion of stationary epithelial cells into migratory, invasive mesenchymal cells via a process known as the epithelial-mesenchymal transition (EMT) [3, 4]. Although brachyury expression was initially reported in embryonic but not adult tissues [2], data mining of human RNA libraries and RT-PCR analysis of multiple tissues recently revealed brachyury to be expressed in a range of human tumors and not in human adult normal tissues, with the exception of testis and a subpopulation of B cells from some healthy donors [5]. Immunohistochemistry studies of human carcinoma biopsy specimens using a brachyury-specific monoclonal antibody (MAb) also demonstrated the expression of brachyury in human lung and breast carcinomas, both in primary tumors and metastases, while limited levels of brachyury were seen in testis and some specimens of thyroid, and no other adult normal tissues examined [6]. High levels of brachyury have also previously been reported in human chordomas [7, 8].

In addition to being essential for embryonic development, recent studies have shown that EMT may also be critical to the progression of carcinomas, as acquisition of mesenchymal features by epithelial tumor cells may favor their dissemination and the acquisition of resistance to various cancer therapies [9, 10]. Recent studies have characterized the transcription factor brachyury as a driver of EMT in human carcinomas [11]. Overexpression of brachyury in human epithelial cancer cell lines led to a more fibroblastoid morphology, a switch from the expression of epithelial markers such as E-cadherin to mesenchymal markers such as N-cadherin, fibronectin and vimentin, and an increase in migration and invasion in *in vitro* assays. Conversely, silencing of brachyury in human tumor cell lines resulted in the loss of mesenchymal features, including loss of migration and invasiveness *in vitro*, and markedly decreased their ability to metastasize in xenograft models [11]. Previous studies have also demonstrated that high levels of brachyury in human carcinoma cells drive resistance to chemotherapy and radiation [12].

While brachyury is a transcription factor of nuclear localization, recent studies have shown that human carcinoma cells can present brachyury peptides in the context of major histocompatibility complex (MHC) class I molecules on the tumor cell surface. This was demonstrated by the identification of a brachyury 9-mer peptide that was used to expand human brachyury-specific CD8^+^ T cells from the blood of cancer patients *in vitro* which, in turn, were able to lyse brachyury-positive tumor cells in an MHC class I–restricted manner [5]. In addition, it has recently been shown that patients receiving a prostate-specific antigen (PSA)–directed vaccine in combination with anti-CTLA4 MAb, or a carcinoembryonic antigen (CEA)–directed vaccine, develop brachyury-specific T cells post-vaccination most likely via the mechanism of antigen cross-presentation [13]. These studies provided evidence of the immunogenicity of brachyury in humans and its potential to serve as a vaccine target.

A previously characterized therapeutic vaccine platform [14-18] consists of heat-killed recombinant *Saccharomyces cerevisiae* (yeast) modified to express tumor-associated antigen(s). For example, a recombinant yeast-CEA vaccine was previously used *in vitro* to efficiently activate murine and human T cells that were lytic against CEA-expressing targets, and *in vivo* for vaccination of tumor-bearing mice resulting in anti-tumor activity. These and other studies have shown that yeast could efficiently activate dendritic cells (DCs) via Toll-like receptors (TLRs) and consequently induce them to produce high levels of type I cytokines, including IL-2, TNF-α, and IFN-γ [14, 16]. The “yeast component” of the recombinant yeast, therefore, is an integral part of the vaccine platform in its ability to activate the innate immune system and might partly contribute to the anti-tumor efficacy of a recombinant yeast construct [15, 17].

In the studies reported here, we have constructed a recombinant *Saccharomyces cerevisiae* (yeast)–brachyury vector-based vaccine (designated as GI-6301), consisting of heat-killed *Saccharomyces cerevisiae* that expresses the full-length human brachyury protein. We report here for the first time that (a) human DCs treated with recombinant yeast-brachyury can activate previously established human brachyury-specific T-cell lines, (b) recombinant yeast-brachyury–treated DCs can expand human brachyury-specific CD8^+^ T cells from peripheral blood of healthy donors and cancer patients, and (c) recombinant yeast-brachyury–treated DCs can expand human brachyury-specific CD4^+^ T cells. It is also shown here that vaccination of mice with recombinant yeast-brachyury can elicit brachyury-specific CD4^+^ and CD8^+^ T-cell responses capable of reducing tumor burden in an experimental model of metastasis. This is accomplished in the absence of any interference with wound healing, or any effect on pregnancy/birth rates and other general toxicology measurements. Based on these results, a Phase I clinical trial of GI-6301 is currently ongoing in patients with advanced tumors [19]; to our knowledge, this is the first vaccine platform aimed at targeting a driver of tumor EMT that has successfully reached the clinical stage.

## RESULTS

### Recombinant yeast-brachyury–treated human DCs activate brachyury-specific human CD8^+^ T cells

Human DCs cultured for 5 days in the presence of recombinant human GM-CSF and IL-4 were incubated for 48 hours with either heat-killed control yeast or heat-killed recombinant yeast-brachyury at a DC-to-yeast ratio of 1:10. Treatment with either construct (control yeast or recombinant yeast-brachyury) resulted in (a) a substantial increase in the percentage of DCs expressing CD80, CD83, and MHC-class I molecules, (b) an increase in the fluorescence intensity of CD86 and MHC-class II molecules, and (c) enhanced production of IL-12, compared to untreated DCs (Supplemental Table 1). It was next examined whether recombinant yeast-brachyury–treated human DCs could efficiently stimulate HLA-A2^+^–restricted brachyury peptide–specific human CD8^+^T cells *in vitro*. DCs generated from peripheral blood mononuclear cells (PBMCs) of two HLA-A2^+^ healthy donors (Table 1, donors 1 and 2) were treated with control yeast or recombinant yeast-brachyury at a DC-to-yeast ratio of 1:1. Forty eight hours after treatment, DCs were irradiated and used to stimulate allogeneic brachyury-specific CD8^+^ T cells that were generated by using a brachyury-specific 9-mer peptide as previously described [5]. As shown in Table 1 with both donors, recombinant yeast-brachyury–treated DCs stimulated brachyury-specific T cells to produce higher levels of IFN-γ than control yeast–treated DCs (approximately 578 vs. 250 pg/ml for donor 1 and 200 vs. 95 pg/ml for donor 2, respectively). The lower level of IFN-γ observed in response to control yeast–treated DCs was in agreement with previous observations [14-16]; as stated above, control (empty) yeast can stimulate DCs via TLRs and the activated DCs, in turn, can activate T cells in a nonspecific manner to secrete type I cytokines.

**Table 1:** Activation of human brachyury-specific CD8^+^ T cells by recombinant yeast-brachyury–treated DCs

	IFN-γ	
	Donor 1	Donor 2
T cells + control yeast–treated DCs	250.8 (± 132.8)	95.2 (± 4.1)
T cells + recombinant yeast-brachyury–treated DCs	577.5 (± 33.2)	200.1 (± 7.2)

DCs generated from PBMCs of 2 HLA-A2^+^ healthy donors were cultured for 7 days in the presence of GM-CSF and IL-4. For the last 48 hours of culture, DCs were treated with control yeast or recombinant yeast-brachyury (DC:yeast ratio of 1:1). DCs were harvested and used to stimulate brachyury-specific CD8^+^ T cells (T cells:DC ratio of 10:1). After 24 hours, culture supernatants were analyzed by ELISA assay for IFN-γ production. Results (pg/ml) represent the mean of replicate measurements (± SD) after subtraction of background with T cells alone.

### Expansion of brachyury-specific CD8^+^ T cells following *in vitro* stimulation with recombinant yeast-brachyury–treated DCs

**Figure 1:**
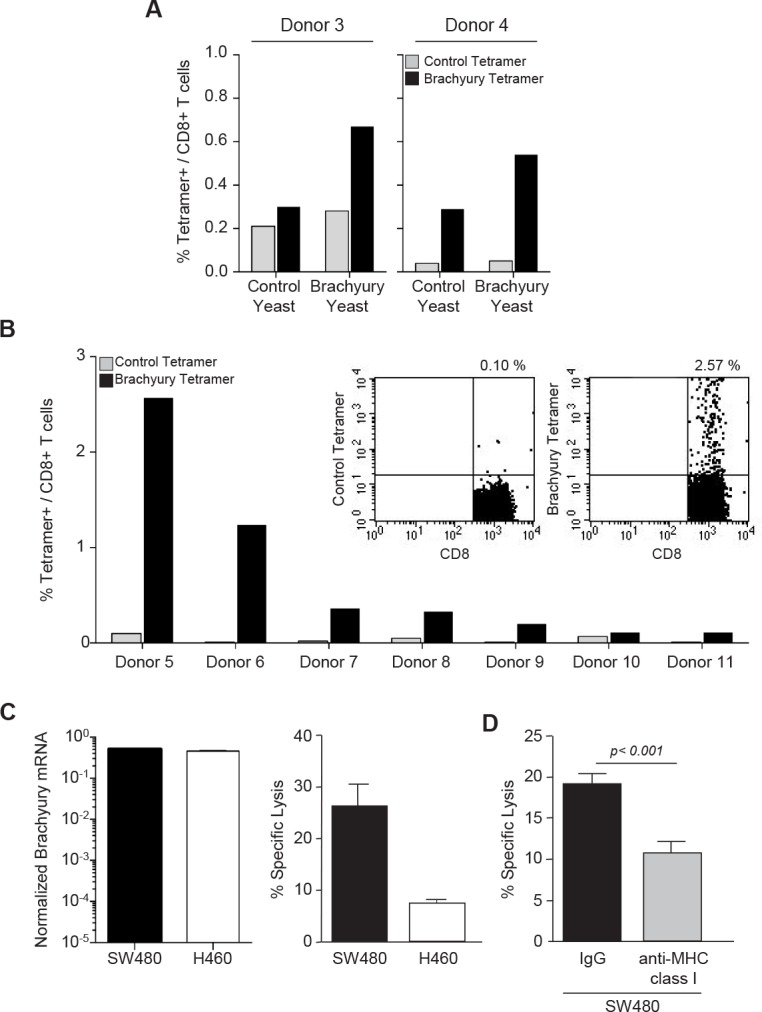
Expansion of brachyury-specific CD8^+^ T cells in response to yeast-brachyury‒treated DCs *A*, Autologous T cells from 2 HLA-A2^+^ healthy donors were stimulated with irradiated control or brachyury yeast‒treated DCs (DC:yeast ratio of 1:1) for 2 IVS cycles. At day 5 of IVS 2, T cells were stained with CD8-FITC and a PE-labeled brachyury-peptide tetramer or a control CMV tetramer. Indicated is the percentage of brachyury (black bars) or control CMV (gray bars) tetramer positive/CD8^+^ T cells as determined by flow cytometry. *B*, Percentage of brachyury or control CMV tetramer positive/CD8^+^ T cells in PBMCs of 7 HLA-A2^+^ healthy donors stimulated for 2 IVS cycles with yeast-brachyury‒treated DCs. Insert shows a representative FACS plot staining for donor 5. *C*, *Brachyury* mRNA expression normalized to *GAPDH* (left panel) and cytotoxic T-cell lysis of SW480 (HLA-A2^+^) and H460 (HLA-A2^neg^) cells with CD8^+^ T cells purified at the end of IVS 2 from donor 5. *D*, A similar CTL assay was conducted with SW480 tumor cells previously incubated with 25 μg/ml of control IgG or anti-MHC class I MAb for 30 min before the addition of brachyury-specific CTLs. The E:T ratio was 60:1. Bars represent the mean of triplicate measurements; error bars represent SEM.

To investigate whether recombinant yeast-brachyury–treated DCs could generate and expand autologous brachyury-specific CD8^+^ T cells from PBMCs, autologous T cells from two HLA-A2^+^ healthy donors (Fig. 1A, donors 3 and 4) were stimulated for two *in vitro* stimulation (IVS) cycles with control yeast– or recombinant yeast-brachyury–treated DCs at a T cell-to-DC ratio of 10:1. At the end of IVS 2, T cells were stained with a PE-labeled brachyury peptide tetramer or a control CMV peptide tetramer. As shown in Figure 1A, the percentage of brachyury tetramer positive/CD8^+^ T cells was higher in cultures stimulated with recombinant yeast-brachyury– compared to control yeast–treated DCs. The detection of some level of brachyury tetramer positive cells in T cells stimulated with control yeast–treated DCs might be attributed, as indicated above, to the ability of control yeast to effectively activate DCs to produce high levels of type I cytokines which, in turn, could induce the nonspecific expansion of some CD8^+^ T cells.

By using PBMCs from seven additional HLA-A2^+^ healthy donors (Fig. 1B, donors 5-11; insert is donor 5) we confirmed these observations; a marked expansion of brachyury tetramer positive/CD8^+^ T cells was observed in 4/7 donors tested at the end of IVS 2. To examine the cytolytic activity of the expanded brachyury-specific CD8^+^ T cells, a cytotoxic T lymphocyte (CTL) assay was performed with T cells expanded from PBMCs of donor 5 after two rounds of IVS with yeast brachyury–treated DCs. CD8^+^ T cells were purified by negative selection using magnetic beads and used as effectors against the human colon carcinoma HLA-A2^+^ SW480 and the lung HLA-A2^neg^ H460 line, which are both positive for brachyury expression (Fig. 1C, left panel). As shown in Figure 1C (right panel), CD8^+^ T cells expanded with recombinant yeast-brachyury–treated DCs preferentially lysed the HLA-A2^+^ SW480 cells, thus indicating the MHC preference of the cytotoxic effect. MHC-restriction was also investigated by performing a lysis assay with SW480 cells pretreated with a control IgG vs. an anti-MHC class I blocking Ab. As shown in Figure 1D, blockade of MHC class I significantly decreased the lysis of SW480 cells, reinforcing the idea that stimulation of PBMCs with recombinant yeast-brachyury–treated DCs induces brachyury-specific CTLs that are capable of lysing brachyury-positive tumor cells in an MHC class I–restricted manner.

**Figure 2:**
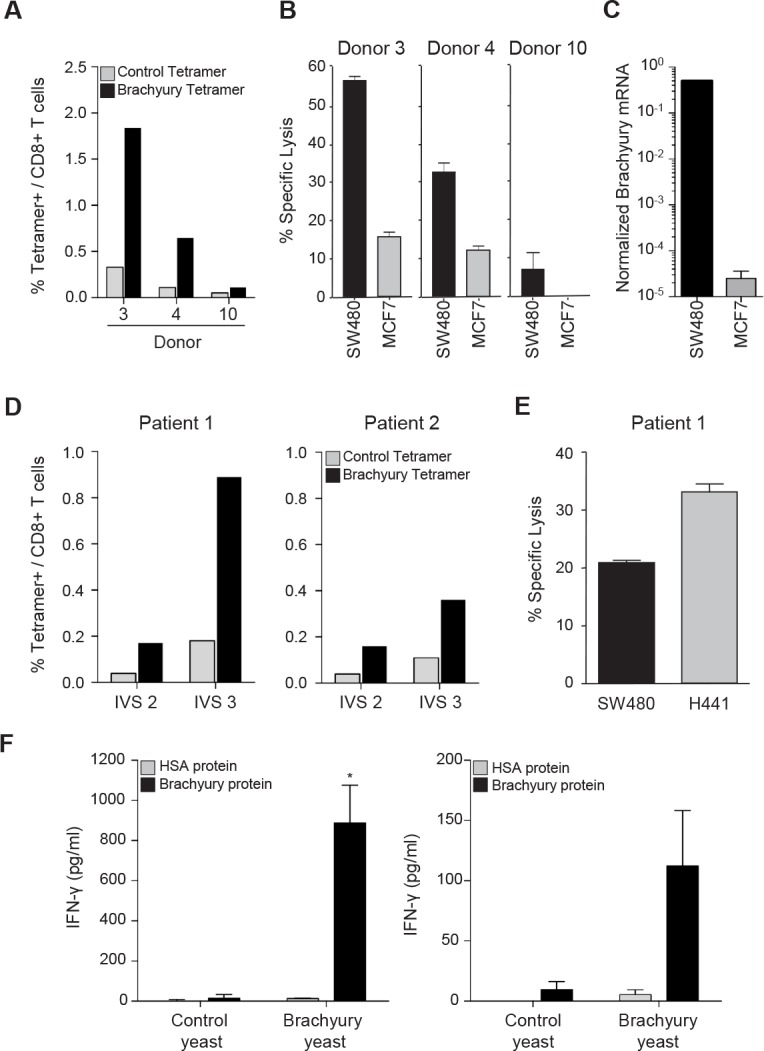
Generation of brachyury-specific CD8^+^ and CD4^+^ T-cell responses upon stimulation with yeast-brachyury‒treated DCs *A*, Autologous T cells from 3 HLA-A2^+^ healthy donors were stimulated with irradiated control yeast‒ or yeast brachyury‒treated DCs (DC:yeast ratio of 1:1) for 2 IVS and then with brachyury-peptide pulsed DCs for an additional IVS cycle (IVS 3). Shown is the percentage of CD8^+^ T cells that co-stained with a brachyury tetramer (black bars) or a control CMV tetramer (gray bars). *B*, On day 6 of IVS 3, CD8^+^ T cells were purified by negative selection and a CTL assay was performed overnight (E:T ratio of 10:1 for donors 3 and 4 and 30:1 for donor 10). ^111^In-labeled HLA-A2^+^ SW480 (brachyury-high) and MCF7 tumor cells (brachyury-low) were used as targets. *C*, *Brachyury* mRNA expression in indicated tumor cells, normalized to *GAPDH*. *D*, Percentage of tetramer positive (brachyury vs. control) in CD8^+^ T cells expanded from blood of 2 breast cancer patients stimulated *in vitro* with yeast-brachyury‒treated DCs for 2 IVS followed by one IVS cycle with brachyury-peptide pulsed DCs. *E*, Purified CD8^+^ T cells from patient 1 were used in a cytotoxic assay with labeled HLA-A2^+^, brachyury-positive SW480 and H441 cells (E:T ratio of 20:1). *F*, Generation of brachyury-specific CD4^+^ T-cell responses upon stimulation with yeast-brachyury‒treated DCs. Autologous T cells from 2 healthy donors were stimulated for 1 IVS cycle (left panel, donor 13), or 2 IVS (right panel, donor 14) with irradiated control or yeast-brachyury‒treated DCs (DC to yeast ratio of 1:1). At the end of IVS 1 (donor 13) or IVS 2 (donor 14), CD4^+^ T cells were purified by negative selection and restimulated with autologous irradiated PBMCs (T cells to APC ratio of 1:3) in the presence of control protein (HSA) or purified His-brachyury protein (10 μg/ml) for 96 hours. Supernatants were analyzed for IFN-γ production by ELISA; results are shown in pg/ml. Bars represent mean of duplicate measurements; error bars represent SD. (*) *p*<0.05.

To further evaluate the ability of recombinant yeast-brachyury–treated DCs to activate brachyury-specific CD8^+^ T cells, T-cell lines were established *in vitro* using recombinant yeast-brachyury–treated DCs for two IVS cycles followed by stimulation with CD40L-activated DCs pulsed with the HLA-A2^+^ brachyury 9-mer peptide Tp2 for an additional IVS cycle, at 10:1 T-cell-to-APC (antigen presenting cells) ratio. On the sixth day of IVS 3, the percentage of brachyury–specific CD8^+^ T cells was determined by tetramer staining. As shown in Figure 2A, a marked expansion of brachyury-specific CD8^+^ T cells was observed in two out of three donors tested (donors 3 and 4). When compared for their cytotoxic activity against tumor targets endogenously expressing brachyury (Fig. 2B), CD8^+^ T cells purified from the PBMCs of donors 3 and 4 were able to efficiently lyse SW480 cells (HLA-A2^+^ and brachyury-high), while lower lysis was observed against the MCF7 breast carcinoma cell line that is HLA-A2^+^ but expresses lower levels of the target brachyury (Fig. 2C). Tumor lysis mediated by purified CD8^+^ T cells was proportional to the percentage of brachyury tetramer CD8^+^ T cells observed for each donor.

Similar studies were conducted to determine whether brachyury-specific T cells could also be expanded from PBMCs obtained from two breast cancer patients. PBMCs from patients 1 and 2 were stimulated for two cycles with recombinant yeast-brachyury–treated DCs (IVS 2, Fig. 2D) followed by one cycle with CD40L-treated DCs pulsed with the Tp2 brachyury peptide (IVS 3, Fig. 2D). About 0.89% and 0.36% of brachyury tetramer positive/CD8^+^ T cells were detected in T-cell cultures from patients 1 and 2, respectively, at the end of IVS 3. CD8^+^ T cells purified from the culture of PBMCs from patient 1 were assayed for cytotoxic activity against the colon SW480 and lung H441 tumor cell lines, both HLA-A2^+^ and brachyury-positive. As shown, brachyury-specific CTLs successfully lysed both tumor cell lines, *in vitro* (Fig. 2E).

### Expansion of CD4^+^ brachyury-specific T cells following stimulation with recombinant yeast-brachyury–treated DCs

The ability of recombinant yeast-brachyury–treated DCs to expand brachyury-specific CD4^+^ T-cell populations was also investigated. PBMCs from nine healthy donors were stimulated in the presence of control yeast– or recombinant yeast-brachyury–treated irradiated DCs (DC-to-yeast ratio of 1:1) for one or two IVS cycles. At the end of each cycle, CD4^+^ T cells were purified by negative selection and restimulated with autologous irradiated PBMCs (T cells-to-APC ratio of 1:3) in the presence of control protein (human serum albumin, HSA) or purified His-brachyury protein. Cell culture supernatants were analyzed for IFN-γ production by ELISA. As depicted in Figure 2F for two different donors, CD4^+^ T cells purified from cultures stimulated with recombinant yeast-brachyury– but not control yeast–treated DCs produced IFN-γ in response to the purified brachyury protein but not control protein. In total, brachyury-specific CD4^+^ T-cell responses were generated from three of nine healthy donors tested. Altogether these results indicated that recombinant yeast-brachyury–treated DCs are capable of expanding both brachyury-specific CD8^+^ and CD4^+^ T cells *in vitro*.

### Immune responses to brachyury elicited by vaccination of mice with yeast-brachyury

**Figure 3:**
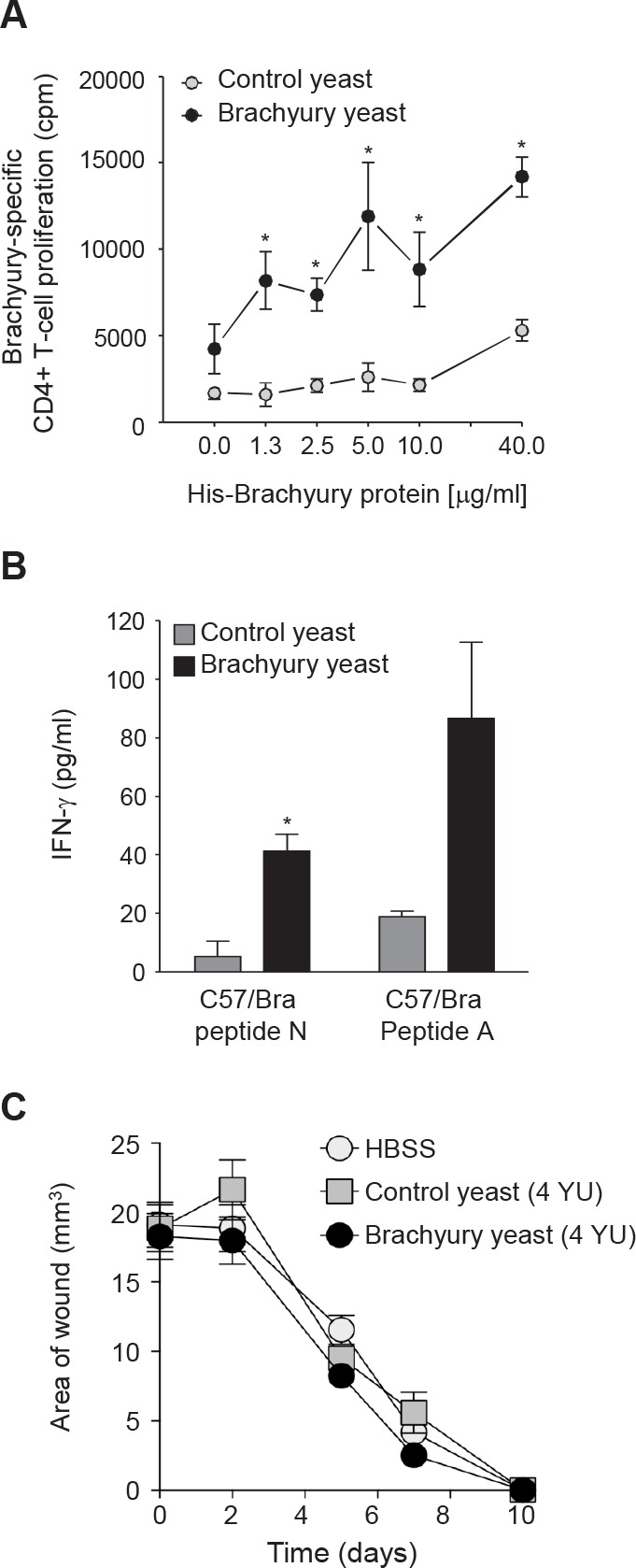
Yeast-brachyury vaccination induces a brachyury-specific T-cell immune response in mice and has no effect on wound healing *A*, A brachyury-specific CD4^+^ T-cell response was observed in animals vaccinated with yeast-brachyury. C57BL/6 mice were vaccinated weekly for a total of 4 vaccinations with 4 YU of yeast-brachyury (black circles) or control yeast (gray circles). Two weeks after the last vaccination, mice were euthanized and purified CD4^+^ T cells were cultured with irradiated APCs and purified His-brachyury protein or β-gal protein for 5 days. ^3^H-thymidine was added (1 μCi/well) for the last 18 hours of culture and proliferation was assayed by measuring incorporated radioactivity. Results were collected in triplicate and expressed as brachyury-specific CD4^+^ T-cell proliferation after background subtraction (proliferation in response to β-gal). (*) *p*<0.05. *B*, Induction of a CD8^+^ T-cell response to brachyury following brachyury-yeast vaccination. C57BL/6 mice were weekly vaccinated for a total of 3 times with 0.1 YU total. Two weeks after the last vaccination, spleens were collected, restimulated with 1 μg/ml of either a C57/Bra peptide N or a C57/Bra peptide A. After 1 week restimulation, CD8^+^ T-cell responses were assayed by IFN-γ production in response to the corresponding stimulating peptide or a control HIV peptide. Supernatants were collected at 48 hours; results represent brachyury-specific IFN-γ production after subtraction of background with HIV. (*) *p*<0.05. *C*, Vaccination with brachyury yeast does not affect wound healing. C57BL/6 female mice were weekly vaccinated for a total of 4 times with 4 YU of brachyury yeast, control yeast or HBSS. One week after the last vaccination, two excisional wounds per mouse were performed as described in Materials and Methods. Shown is the average of the wound area (n=4 mice per group) at various time points.

We then evaluated whether vaccination with yeast brachyury could elicit a brachyury-specific immune response. Brachyury is somewhat conserved between human and mice, with a 91% identical amino acid sequence; therefore, vaccination with recombinant yeast human brachyury would be expected to elicit an immune response against the murine homolog. C57BL/6 mice were vaccinated with 1 YU(yeast unit) control or recombinant yeast-brachyury injected subcutaneously (s.c.) at four different sites (total 4 YU per mice) on days 0, 7, 14 and 21. Mice were euthanized 2 weeks following the final boost and CD4^+^ T-cell proliferation was performed employing both purified His-brachyury and β-gal control protein (background correction). As shown in Figure 3A, vaccination with yeast-brachyury induced significant proliferation of CD4^+^ T cells at all concentrations of brachyury protein, compared to control yeast.

The effect of brachyury vaccination on CD8^+^ T-cell immune responses in C57BL/6 mice was also evaluated. Mice were injected (s.c.) with 0.025 YU per site at four sites (0.1 YU total per mice) on days 0, 7, and 14, and euthanized 2 weeks after the final boost. Splenocytes were cultured for 1 week in the presence of 1 μg/ml of the brachyury peptides N or A (see Materials and Methods), and restimulated on day 7 with APCs pulsed with 1 μg/ml of the corresponding brachyury peptides or a control HIV peptide. Supernatants were evaluated for IFN-γ. As shown in Figure 3B, vaccination with yeast brachyury, but not control yeast, expanded brachyury-specific CD8^+^ T cells to both peptides.

### Brachyury vaccination does not impair wound healing

Because of the role of brachyury during EMT [11, 20] and the recognized role of EMT during the process of wound healing and tissue regeneration, we sought to investigate whether mice vaccinated with yeast-brachyury could have an impaired ability to heal experimentally induced wounds. C57BL/6 mice were vaccinated weekly with HBSS, 4 YU of yeast-brachyury or 4 YU of control yeast injected at four subcutaneous sites at 1 YU per site. Two weeks after the last vaccination, excisional wounds were made in the flank of the animals by skin punch biopsy and were subsequently measured (two diameters) on days 2, 5, 7, and 10 and the surface area calculated. As shown in Figure 3C, comparable wound healing occurred in all groups over the 10-day observation period following the insult. Complete wound closure was observed in all animals, regardless of the vaccination received.

### Antitumor effect of recombinant yeast-brachyury vaccination

**Figure 4:**
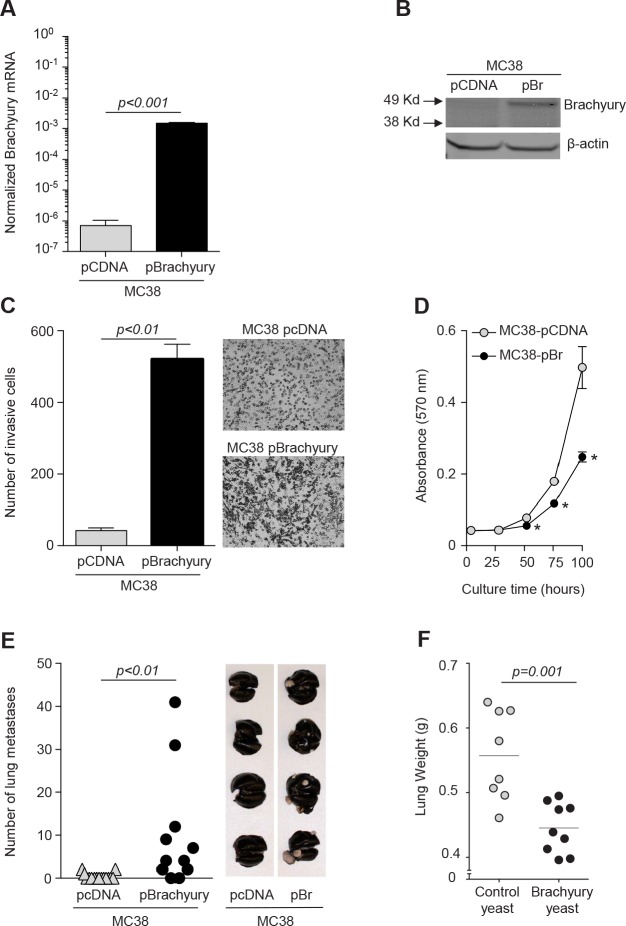
Antitumor effect of yeast-brachyury vaccination in the MC38-brachyury metastatic tumor model *Brachyury* mRNA (*A*) and protein (*B*) expression levels in MC38 cells stably transfected with either an empty vector (pcDNA) or a vector encoding the full-length human *brachyury* gene (pBrachyury). *C*, *In vitro* ECM invasion assay for MC38-pCDNA and MC38-pBrachyury cells. Results of 1 of 2 experiments are shown in the graph and representative images of the bottom of the filters are also shown. *D*, *In vitro* proliferation of MC38-pCDNA and MC38-pBrachyury cells as assessed by the MTT assay. *E*, One million tumor cells (MC38-pCDNA or MC38-pBrachyury) were injected intravenously via tail vein on day 0 into C57BL/6 mice. At sacrifice (day 40), lungs were inflated with India Ink and the number of lung tumor nodules enumerated. Representative images of lungs in each group are displayed. *F*, MC38-pBrachyury tumor cells (1 × 10^6^ cells) were implanted in C57BL/6 mice via tail vein on day 0. Mice were subsequently vaccinated on day 4 with brachyury-yeast or control-yeast (1 YU × 4 sites, weekly boosters). At sacrifice, lungs were inflated with India ink and weighed. In all graphs, error bars indicate the standard error of the mean (SEM) of triplicate measurements.

While brachyury is expressed in a wide range of human tumors, its expression, to our knowledge, in murine tumors is limited to embryonal carcinoma cell lines [21] with mixed genetic background or no MHC class I expression, which precludes their use in immunotherapeutic studies. To employ a more controlled experimental condition, the murine colon MC38 murine carcinoma cell line was stably transfected with a control empty vector or a vector encoding the full-length human brachyury protein (Fig. 4A, B). In agreement with our previous observations on the role of brachyury in EMT in human carcinoma cell lines, this genetic manipulation of MC38 cells resulted in a dramatic increase in their ability to invade the extracellular matrix (ECM), as indicated by the results of an *in vitro* invasion assay shown in Figure 4C. Also in agreement with our previous studies conducted with human tumor cells [12], the overexpression of brachyury significantly decreased, rather than increasing, the proliferation of MC38 tumor cells *in vitro* (Fig. 4D). *In vivo*, MC38 cells with or without brachyury grew at similar rates as subcutaneous tumors (data not shown); however, the ability to form experimental metastasis after direct intravenous injection was significantly enhanced in MC38-pBrachyury as compared to MC38-pcDNA cells (Fig. 4E). These results thus reinforced the concept that brachyury is a relevant protein during tumor progression by promoting the acquisition of a tumor invasive phenotype and enhancing metastatic propensity.

The potential anti-tumor effect of recombinant yeast-brachyury vaccination was evaluated in C57BL/6 mice that were intravenously injected with MC38-pBrachyury cells on day 0 and subsequently vaccinated weekly from day 4 with 1 YU per site at four sites with recombinant yeast-brachyury or the control yeast vaccine, until sacrifice on day 36. Analysis of lung weight revealed a significant reduction (*p*=0.001) in the weight of the lungs in the recombinant yeast-brachyury vs. control yeast vaccinated group (Fig. 4F). A reduction in the number of lung metastases among animals vaccinated with recombinant yeast-brachyury vs. control yeast was also observed, with 1/9 (11.1%) vs. 4/8 (50%) animals having ≥ 5 visible lung metastasis in each group, respectively.

### Immune responses to brachyury in the absence of toxicity

Although brachyury expression in normal murine tissues is limited [21], the potential for toxicity elicited by brachyury-specific T cells against normal tissues was evaluated in a murine model. Balb/c mice were vaccinated weekly with control yeast or recombinant yeast-brachyury (1 YU/site, 4 sites), or HBSS for 18 weeks. Mice were monitored daily for clinical signs such as respiratory distress and morbidity, and weighed weekly. One week following the final vaccination, mice were euthanized and analyzed for autoantibodies in serum, blood chemistry parameters, and pathology of major tissues. During the course of vaccination with recombinant yeast-brachyury, no mice exhibited any signs of morbidity or respiratory distress. In addition, mice vaccinated with recombinant yeast-brachyury, control yeast or HBSS showed no significant weight decreases during the course of the study and no changes in blood counts, serum chemistry, autoimmune panels or histopathology were observed in any of the groups (Table 2A). Similar results were obtained in a parallel study conducted with C57BL/6 mice (data not shown).

**Table 2A:** Recombinant yeast-brachyury vaccination does not induce toxicity

	HBSS	Control yeast	Brachyury yeast
Body weight (normal ranges 18-26 g)	25.3 ± 1.7	24.5 ± 2.2	25.3 ± 2.1
CBC (n=20)	Normal	Normal	Normal
Serum chemistry (n=7)	Normal	Normal	Normal
Autoimmune panel (n=5)	Normal	Normal	Normal
Histopathology (n=10)	Normal	Normal	Normal

Groups of 5 female Balb/c mice were vaccinated with HBSS, control yeast, or recombinant yeast-brachyury (1 YU per site at 4 sites) as described in the Materials and Methods section. One week after the last vaccination, animals were weighed, euthanized and tissues collected for evaluation.

Because of the role of brachyury during embryonic development, it was also investigated whether vaccination with recombinant yeast-brachyury could have any effect on subsequent pregnancies or the condition of the offspring. Female C57BL/6 mice were vaccinated with HBSS or control yeast or recombinant yeast-brachyury (1 YU per site at four sites) weekly for a period of 4 weeks. Two weeks after the last vaccination, females were bred and the rate of pregnancy, litter size, general condition of the pups, as well as the development of the pups post-weaning were evaluated for each group. As shown in Table 2B, brachyury-yeast vaccination had no effect in any of the parameters evaluated.

**Table 2B:** Effect of recombinant yeast-brachyury vaccination on future pregnancies

	HBSS	Control yeast	Brachyury yeast
Rate of pregnancy (%)	8/10 (80%)	7/10 (70%)	7/10 (70%)
Number of pups (range per mother)	54 (5-9)	52 (3-11)	51 (5-9)
Condition of the newborns	Normal	Normal	Normal
Condition of the pups at weaning	Normal	Normal	Normal
Number of mice at endpoint	27	36	34

Groups of 10 female C57BL/6 mice were vaccinated with HBSS, control yeast, or recombinant yeast-brachyury (1 YU per site at 4 sites) weekly for a period of 4 weeks. Two weeks after the last vaccination, females in each group were bred (harem-breeding was performed). Rate of pregnancy, litter size, general condition of the pups, as well as development of the pups post-weaning were evaluated for each group.

## DISCUSSION

The phenomenon of tumor EMT is now thought to be critical to the progression of carcinomas by promoting the dissemination of cancer cells and the acquisition of mechanisms of resistance to various cancer therapies [4]. This study reports for the first time the development and characterization of an immunological approach to potentially targeting tumor cells undergoing EMT via the use of a recombinant yeast vaccine that expresses brachyury, a transcription factor able to drive EMT in human carcinomas [11, 12, 20]. We demonstrate here that a recombinant yeast-brachyury vaccine can activate and expand brachyury-specific human CD8^+^ and CD4^+^ T cells *in vitro* from the blood of healthy donors and cancer patients, and elicits an effective immune response *in vivo* in vaccinated mice, which drives anti-tumor activity in the absence of toxicity.

The approach described here could potentially be applied towards the treatment of several types of cancer. Expression of the brachyury protein has been previously demonstrated with a polyclonal antibody in chordomas [7, 8]. In subsequent studies employing RT-PCR [5, 11, 21] or monoclonal antibodies in western blot or immunohistochemistry [6], the expression of brachyury was also demonstrated in a range of human carcinomas, including breast, lung, colorectal, pancreatic, and ovarian, and not in the majority of normal tissues evaluated. In many cases, brachyury was only expressed in a minority of primary tumor cells while a higher proportion of brachyury-positive tumor cells and higher levels of expression were observed in metastatic lymph nodes or distal metastases [6]. This is not surprising since the EMT process is thought to be dynamic and the transition from an epithelial to a mesenchymal phenotype can be influenced by factors in the tumor microenvironment, such as IL-8 and TGF-β [20, 22]. In addition to carcinomas, brachyury is also expressed in multiple myeloma and chronic lymphocytic leukemia cells (unpublished data). Recent data has also shown that brachyury is an independent poor prognostic indicator in lung cancer [23], early colorectal cancer [24], and breast cancer (unpublished data).

Multiple studies have now demonstrated a relationship between EMT and tumor “stemness” [25-27]. Both “cancer stem-like” cells and tumor cells undergoing EMT are characterized by drug resistance [10, 28], and are known to overexpress several transcription factors, including OCT4 and SOX2, that are involved in the control of cell pluripotency [27, 29]. Thus, targeting of tumor cells undergoing EMT could also result in the elimination of cancer cells that exhibit tumor-initiating properties. Up to date, several transcription factors have been identified that are able to drive tumor EMT; one distinction that has been reported between brachyury and others, incuding TWIST1, SNAIL and SLUG, is the more selective expression of brachyury in tumors vs. normal tissues [6, 21], which makes it an attractive target for anti-tumor interventions. Transcription factors, however, are thought to be “undruggable” by direct, conventional small molecule inhibitor approaches due to their lack of a specific groove for tight binding of an inhibitor. In addition, their intracellular localization would preclude the use of monoclonal antibody targeting approaches. The use of a vaccine therapy that relies on T-cell mediated targeting of the transcription factor may circumvent these characteristics. Although transcription factors, such as brachyury, would be expected to be found only in the nuclear compartment, short fragments of brachyury (9-15 amino acids long) are processed and transported to the surface of the tumor cell in the context of MHC class I and class II molecules to activate brachyury-specific CD8^+^ and CD4^+^ T cells, respectively. As shown here, DCs treated with recombinant yeast-brachyury can indeed generate both human CD4^+^ and CD8^+^ T-cell responses specific for brachyury. The murine studies reported here also show that the recombinant yeast-brachyury can elicit both CD8^+^ and CD4^+^ T-cell responses *in vivo* as well as anti-tumor activity.

A vaccine consisting of a recombinant yeast-brachyury has some potential favorable characteristics: recombinant yeast is relatively easy to generate, can be propagated and stored as an “off-the-shelf” drug, and is heat-killed and thus relatively safe [30, 31], as shown by an excellent safety profile in prior clinical studies of recombinant yeast vaccines directed against hepatitis C virus, mutated *ras* and CEA. In addition, the recombinant yeast-brachyury contains the full-length human brachyury gene product and thus could be able to elicit both CD4^+^ and CD8^+^ T-cell responses regardless of the MHC allele(s) of the patient. While the recombinant yeast-brachyury vaccine is shown here to be more efficacious than the control yeast in the induction of brachyury-specific immune responses *in vitro* and *in vivo* and anti-tumor activity, the experiments reveal some degree of immune effect with control yeast. Previous studies have shown that control yeast can induce a strong innate immune response; treatment of murine or human DCs with control yeast efficiently matured DCs and induced secretion of type I cytokines to similar levels than those elicited by TLR agonists. Thus it is confounding to consider the control yeast vector as a true control for any immune response.

Toxicology studies are important when dealing with a target involved in EMT as this process is also implicated in tissue remodeling and wound healing. Here we have conducted toxicology studies in mice and reported no toxicities associated with yeast-brachyury vaccination. The murine toxicology studies, however, cannot substitute for a carefully designed incremental dosing Phase I trial; such trial has already been initiated employing GI-6301 in patients with metastatic cancer [19]. To our knowledge, this is the first vaccine platform aimed at targeting a driver of tumor EMT that has successfully reached the clinical stage.

## MATERIALS AND METHODS

### Culture and maturation of DCs

Peripheral blood used in this study was collected from healthy donors and cancer patients after Institutional Review Board approval and informed consent were obtained. PBMCs were isolated from peripheral blood by centrifugation on a Ficoll density gradient. For the preparation of DCs, PBMCs were resuspended in AIM-V medium (Invitrogen, Carlsbad, CA) and allowed to adhere to the surface of T-150 flasks for 2 hours at 37°C; the adherent cells were cultured for 7 days in AIM-V medium containing recombinant human GM-CSF (100 ng/ml) and IL-4 (20 ng/ml) (Peprotech, Rocky Hill, NJ). For maturation of DCs, control yeast or a recombinant yeast-brachyury preparation at a ratio of yeast-to-DCs of 1:1, or 0.5 μg/ml recombinant human CD40 ligand (CD40L) plus 1 μg/ml cross-linker enhancer (Enzo Life Sciences, Farmingdale, NY) were added to the cultures 48 hours prior to use.

### Human tumor cell lines

The human tumor cell lines used in this study, colon SW480, breast MCF7, and lung H460 and H441, were purchased from the American Type Culture Collection (ATCC, Gaithersburg, MD) and propagated as recommended by the ATCC.

### Yeast vectors

Two recombinant *Saccharomyces cerevisiae* constructs were utilized in this study: a control empty construct (designated as control yeast) and a construct expressing the full-length human brachyury protein (designated as recombinant yeast-brachyury, GI-6301). The expression of brachyury in the recombinant yeast-brachyury vaccine, as detected with an anti-brachyury murine MAb, is shown in Supplemental Figure 1. These vectors were engineered as previously described [32] as part of a Cooperative Research and Development Agreement (CRADA) between the Laboratory of Tumor Immunology and Biology, NCI, and GlobeImmune, Inc. (Louisville, CO).

### Peptides and proteins

The human brachyury-specific HLA-A2 binding 9-mer peptide used in this study (designated as peptide Tp2, WLLPGTSTL) was previously described [5]. Two peptides derived from the sequence of the human brachyury protein were used in experiments with C57BL/6 mice, the C57/Bra peptide N (WSVSNGAVT) and the C57/Bra peptide A (WSVSNGAVL). To assess CD4^+^ T-cell responses, a His-brachyury fusion protein was produced via a baculovirus expression system in insect cells. HSA protein was used as control (Sigma, St. Louis, MO).

### Expansion of brachyury-specific CD8^+^ T cells *in vitro*

Brachyury-specific cytotoxic T lymphocytes were generated from the blood of healthy donors or two different breast cancer patients post-vaccination with a MUC1-CEA-TRICOM-based vaccine. Briefly, yeast-treated or peptide-pulsed irradiated (30 Gy) DCs were used as antigen-presenting cells (APCs) with autologous PBMCs, used at a ratio of PBMC-to-APC of 10:1. Cultures were maintained for 3 initial days in RPMI 1640 medium (Mediatech, Inc., Herndon, VA) supplemented with 10% human AB serum (Gemini Bio-Products, West Sacramento, CA), and for 4 additional days in the same medium supplemented with 20 U/ml of recombinant human IL-2. After a 7-day culture period, designated as an *in vitro* stimulation (IVS) cycle, cells were re-stimulated as described above.

### Expansion of brachyury-specific CD4^+^ T cells *in vitro*

Control yeast or recombinant yeast-brachyury–treated DCs were irradiated (30 Gy) and used as APCs to stimulate autologous PBMCs *in vitro* at a PBMC-to-APC ratio of 10:1. Cultures were maintained as above. At the end of one to two *in vitro* IVS, CD4^+^ T cells were isolated by negative selection with magnetic beads (Miltenyi Biotec, Auburn, CA) and re-stimulated (1 × 10^4^ CD4^+^ T cells per well) with autologous irradiated PBMCs as APCs (CD4-to-APC ratio=1:3), in the presence of either HSA (control protein) or His-brachyury protein (10 μg/ml) for 96 hours. Cell culture supernatants were collected; IFN-γ was measured by ELISA.

### Flow cytometry

PE-labeled anti-CD80, anti-CD83, anti-CD86, anti-HLA Class I, anti-HLA Class II, and appropriate isotype controls were purchased from BD Biosciences (San Diego, CA). A PE-conjugated HLA-A2^+^/Tp2 brachyury peptide tetramer (NIH Tetramer Core Facility, Atlanta, GA) and a control HLA-A2^+^/CMV (NLVPMVATV) tetramer (Beckman-Coulter, Brea, CA) were used.

### Detection of cytokines

Culture supernatants were collected at indicated times and analyzed by ELISA for the presence of IL-12 (BD Biosciences) or IFN-γ (Invitrogen). Results were expressed in pg/ml.

### Cytotoxic T-cell killing assay

Target cells were labeled with 50 μCi of ^111^Indium-labeled oxyquinoline (Amersham Health, Silver Spring, MD) and plated (2 × 10^3^ cells per well) with effector cells at various effector-to-target (E:T) cell ratios. For MHC class I blockade experiments, labeled target cells were incubated with 25 μg/ml anti-human HLA-ABC antibody (Serotec, Raleigh, NC) or IgG1 isotype control (BD Biosciences) for 30 min at 37°C, before the addition of effector cells. Supernatants were harvested after 16 hours and ^111^In release was measured by gamma counting.

### Brachyury gene expression

RNA from human and murine carcinoma cells was purified using the Qiagen RNeasy kit (Valencia, CA). Real-time PCR was performed according to the manufacturer's protocol using 10 ng cDNA and the following TaqMan gene expression assays from Applied Biosystems: brachyury (Hs_00610080), human and murine GAPDH (4326317E, 4352339E).

### Animals

All mice were housed and maintained in microisolator cages under specific pathogen-free conditions and in accordance with the Association for Assessment and Accreditation of Laboratory Animal Care (AAALAC) guidelines. All experimental studies were carried out under approval of the NIH Intramural Animal Care and Use Committee.

### Murine CD4^+^ and CD8^+^ T-cell responses

C57BL/6 mice were vaccinated weekly with either yeast control or recombinant yeast-brachyury vaccine at 1 YU per site at four sites (4 YU total) for 4 weeks. Fourteen days after the last vaccination, animals were euthanized, splenocytes collected and CD4^+^ T cells purified by negative selection (Miltenyi Biotec). CD4^+^ T cells (1 × 10^5^) were plated with 5 × 10^5^ irradiated syngeneic spleen cells and indicated concentrations of either His-brachyury or β-galactosidase (β-gal) protein. After 4 days of incubation, 1 μCi of [^3^H]-thymidine (Perkin-Elmer, Waltham, MA) was added per well; plates were harvested after 16 hours and thymidine incorporation was measured using a 1450 Betaplate reader (Perkin-Elmer). Proliferation was corrected for β-gal counts. For CD8^+^ T-cell responses, mice were vaccinated weekly with either vaccine at 0.025 YU per site at 4 sites (0.1 YU total) for 3 weeks, followed by 2 weeks rest, and splenocytes were collected and stimulated for 6 days with 1 μg/ml of C57/Bra peptide N or peptide A. T cells (1 × 10^5^) were cultured with 5 × 10^5^ irradiated syngeneic spleen cells along with 1 μg/ml of the brachyury or a control HIV gag peptide (SQVTNPANI). Culture supernatants were collected at 24 hours; IFN-γ was assessed by ELISA. Results were background-corrected for HIV peptide.

### Effect of yeast-brachyury vaccination on wound healing

Groups of five female C57BL/6 mice were vaccinated with 1 YU at four sites (4 YU total) of yeast control or recombinant yeast-brachyury weekly for 4 weeks. One week after the last injection animals were anesthetized, shaved and wounded utilizing a 4 mm diameter AcuPunch skin biopsy punch (Acuderm Inc, Ft. Lauderdale, FL). Wounded areas were measured until closure.

### Effect of yeast-brachyury vaccination on future pregnancy

Groups of 10 female C57BL/6 mice were weekly vaccinated with yeast control or yeast-brachyury at 1 YU per site at four sites (4 YU total) for 4 weeks. An additional group of 10 control animals were similarly injected with Hank's balanced solution (HBSS). One week after the last injection, animals were placed for harem breeding. The rate of pregnancy, litter size, as well as the general condition of the pups were evaluated.

### Toxicology study

Groups of five female BALB/c mice were vaccinated with 1 YU per site at four sites (4 YU total) of yeast control or yeast-brachyury weekly for 12 weeks, then every 2 weeks for an additional 8 weeks, and subsequently every month for 2 months. An additional group of 10 control animals was similarly injected with HBSS. One week following the last injection, animals were assessed for any potential toxicities in a blinded manner at Charles River Pathology Associates (Wilmington, MA) utilizing the following parameters: in-life body weight, histopathology (10 tissues), serum chemistry (7 parameters), CBC (20 parameters), and autoimmunity (5 parameters) as described previously [33].

### Tumor cell transfection

Murine colon carcinoma MC38 cells were stably transfected with an empty pcDNA4/TO vector (Invitrogen, designated as pcDNA) or a full-length human brachyury-encoding (pBrachyury) vector using a Nucleofector system according to the manufacturer's recommendations (Lonza, Walkersville, MD). Transfected cells were selected in media containing 100 μg/ml Zeocine (Invitrogen).

### Tumor cell migration, invasion and growth assays

Migration and invasion assays were performed as previously described [11]. RPMI 1640 supplemented with 20% FBS medium was added to the lower chambers and 1 × 10^5^ cells in serum-free medium were added onto the upper chambers, followed by incubation overnight at 37°C. Cell growth was evaluated using 3-4,5-dimethylthiozol-2, 5-diphenyl-tetrazolium bromide (MTT) (Sigma-Aldrich, St. Louis, MO).

### Experimental model of metastasis and antitumor vaccination

C57BL/6 mice were injected with 1 × 10^6^ MC38-pCDNA (n=15) or MC38–pBrachyury (n=11) cells intravenously into the tail vein. Forty days after tumor implantation animals were euthanized, lungs were inflated with India ink and tumor nodules were counted utilizing a dissecting microscope. For antitumor experiments, mice were injected with 1 × 10^6^ MC38-pBrachyury cells intravenously into the tail vein; 4 days after tumor implantation, groups of mice were vaccinated with either control yeast (n=8) or recombinant yeast-brachyury (n=9) at a dose of 1 YU at four sites (4 YU total) and subsequently weekly for the duration of the experiment. Mice were euthanized on day 36 post-tumor implantation; lungs were inflated with India Ink, visually inspected for the presence of tumor nodules and weighed.

### Statistical analysis

Data were analyzed using GraphPad Prism (Version4; GraphPad Software). Data points in graphs represent the mean of triplicate measurements ± standard error of the mean (SEM), unless indicated.

## Supplementary Figure and Table




