# Supplementary material for: Immunological targeting of tumor cells undergoing an epithelial-mesenchymal transition via a recombinant brachyury-yeast vaccine
Source: Oncotarget. 2013 Sep 26;4(10):1777–90. doi: 10.18632/oncotarget.1295 (PMC3858563; doi:10.18632/oncotarget.1295)
Supplement: Supplementary file 1 [file oncotarget-04-1777-s001.pdf]

Immunological targeting of tumor cells undergoing an epithelial-mesenchymal transition via a recombinant brachyury-yeast vaccine - Hamilton et al

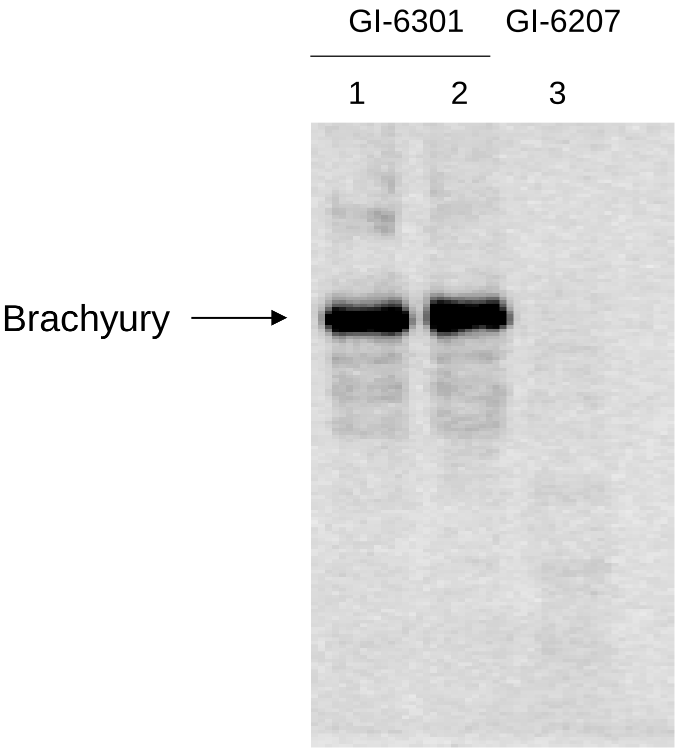

Supplemental Figure 1: Expression of Brachyury in recombinant yeast-Brachyury (GI-6301). Brachyury yeast were cultured in UL2 medium to mid-exponential phase, and Brachyury antigen expression was induced with 375 μM $CuSO_4$ for 3 hours. The culture was harvested, PBS-washed, and heat-inactivated at 56º C for 1 hour. Twenty YU (20 x 10^7 yeast cells) of yeast cells was lysed by glass bead rupture and heating for 5 minutes in denaturing SDS buffer (95ºC). Total protein was quantified using a TCA precipitation/nitrocellulose binding assay and 0.2 μg was fractionated by SDS-PAGE. Detection and quantification of the his-tagged Brachyury antigen was done by Western blot using a mouse monoclonal anti-Brachyury antibody (Abcam clone 5H8; lanes 1 and 2). GI-6207 is a lysate of yeast that express his-tagged CEA (used as specificity control, lane 3).
