# Supplementary material for: Immunological targeting of tumor cells undergoing an epithelial-mesenchymal transition via a recombinant brachyury-yeast vaccine
Source: Oncotarget. 2013 Sep 26;4(10):1777–90. doi: 10.18632/oncotarget.1295 (PMC3858563; doi:10.18632/oncotarget.1295)
Supplement: Supplementary file 2 [file oncotarget-04-1777-s002.pdf]

Immunological targeting of tumor cells undergoing an epithelial-mesenchymal transition via a recombinant brachyury-yeast vaccine - Hamilton et al

Supplemental Table 1: Expression of activation markers by yeast-treated DCs

Dendritic Cells	CD80 % (MFI)	CD83 % (MFI)	CD86 % (MFI)	Class I % (MFI)	Class II % (MFI)	IL-12
Untreated	5.0 (26)	7.4 (30)	97.9 (156)	76.6 (93)	97.3 (112)	73.8 (±14.8)
Control Yeast	16.5 (54)	42.6 (49)	98.8 (268)	91.5 (97)	98.0 (194)	2419.0 (±54.2)
Brachyury Yeast	35.0 (56)	44.1 (40)	99.4 (331)	93.9 (92)	98.4 (174)	1839.8 (±409.0)

DCs generated from healthy donor PBMCs were cultured for 7 days in the presence of GM-CSF and IL-4. For the last 48 hours of the culture, DCs were left untreated or were treated with control yeast or recombinant yeast-Brachyury (DC:yeast ratio of 1:10). Culture supernatants were collected and analyzed for IL-12 levels by using an ELISA kit (BD Biosciences). Results (pg/ml) represent the mean of replicate measurements (± SD). DCs were harvested and expression of surface molecules was analyzed by FACS analysis as described in the Materials and Methods section.
